# Direct puncture of an occluded common femoral artery as a new approach for endovascular aortic aneurysm repair

**DOI:** 10.1186/s42155-021-00247-1

**Published:** 2021-07-10

**Authors:** Shigeo Ichihashi, Tsunehisa Yamamoto, Francesco Bolstad, Keitarou Koshi

**Affiliations:** 1 Department of Radiology, Nara Medical University, 840 Shijyocho, Kashihara, Nara, 634-8521 Japan; 2 Department of Cardiovascular Surgery, Kyoto Okamoto Memorial Hospital, 100 Nishinokuchi, Kumiyama, Kuse District, Kyoto, 613-0034 Japan; 3 Department of Clinical English, Nara Medical University, 840 Shijyocho, Kashihara, Nara, 634-8521 Japan

**Keywords:** Abdominal aortic aneurysm, Direct puncture, Endovascular procedures, Common femoral artery, Endarterectomy

## Abstract

**Background:**

Abdominal aortic aneurysms (AAA) which present with a hostile access are not uncommon. When an arterial occlusion continuously involves from the iliac to the femoropopliteal artery, endovascular aneurysm repair (EVAR) can become complex, necessitating an adjunctive surgical procedure. The present report outlines a successful EVAR which was conducted without any adjunctive surgical procedure for an AAA complicated by extensive access vessel occlusion.

**Case presentation:**

The patient, an 82-year-old male, had a fusiform 50 mm infrarenal AAA. He had a history of above knee amputation of the right leg due to a gangrene from Buerger’s Disease. Despite the continuous occlusions of the right external iliac artery (EIA), common femoral artery (CFA), and superficial femoral and profunda femoris artery, limb ischemia was not observed in his right leg. Since revascularization of the occluded right iliac and femoral arteries was deemed to be too complex technically and no ischemic symptoms were observed, EVAR was performed using the occluded access only for the delivery of the stent graft without restoring the flow. Firstly, the occluded right CFA was punctured under ultrasound guidance. Next, a 0.014 in. guidewire and a microcatheter were successfully navigated to the subintimal space of the right common iliac artery (CIA), these were then exchanged with a reentry device. The reentry device allowed the advancement of a guidewire into the true lumen of the right CIA. Then, a 12Fr sheath for delivery of a contralateral limb was advanced via the occluded right access to aorta, and a 16 Fr sheath for delivery of a main body graft was advanced via a patent left iliac artery. A standard EVAR procedure was subsequently performed.

**Conclusions:**

EVAR was successfully performed for an AAA complicated with an arterial occlusion from the EIA to the SFA using direct puncture of the occluded CFA. This technique could be an effective measure for cases with a hostile access involving the CFA.

## Background

Hostile iliac access is an obstacle to perform endovascular aneurysm repair (EVAR), and it reportedly precludes EVAR in 6–15.4% of patients with abdominal aortic aneurysms (AAA) (Arko et al. 2004). These occlusive iliac accesses are manageable in most cases by endovascular revascularization alone or combining surgical procedures such as creating iliac conduit for delivery of the endograft (Uchiyamada et al. 2013; Vallabhaneni et al. 2012; Takeuchi et al. 2019; Giannopoulos et al. 2021). However, in cases where the occlusion of the iliac artery extends to the common femoral artery (CFA), a concomitant surgical intervention such as endarterectomy of the CFA is also necessary. Such revascularization procedures are technically demanding in cases where runoff, femoropopliteal vessels are completely occluded. The present report describes a case of AAA with a coexisting arterial occlusion ranging from the external iliac artery (EIA) to the superficial femoral artery (SFA). In this case, a direct puncture of the occluded CFA was successfully employed to navigate the endograft via the occluded iliac artery.

## Case presentation

**Fig. 1:**
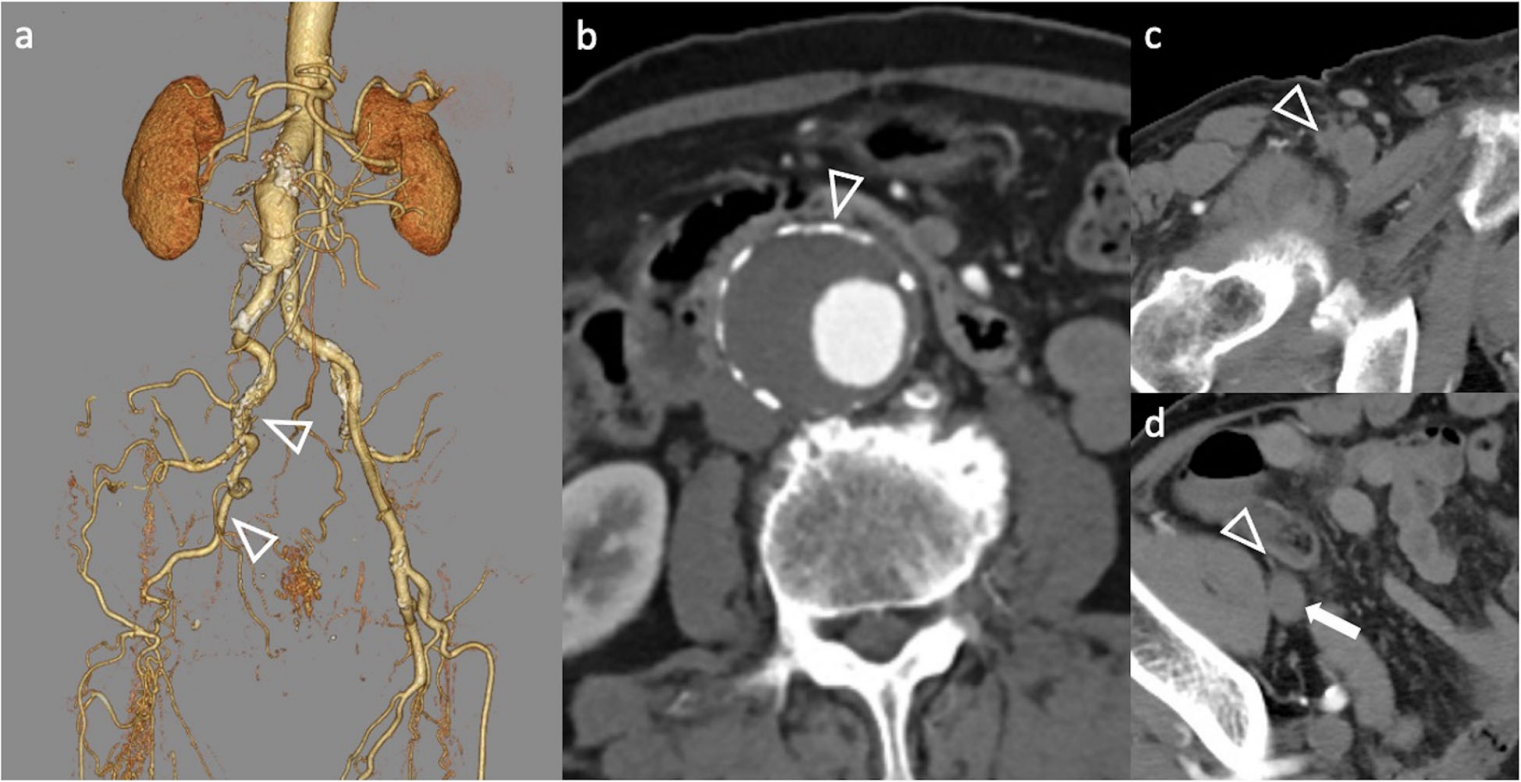
CT before endovascular aneurysm repair. **a** VR image of CT angiography shows the occlusion of the right EIA to the SFA with collateral network from the right hypogastric artery (arrow heads), **b** Axial plane of CT shows AAA (arrow head), **c** Contrast enhanced CT shows occlusion of the right CFA (arrow head), **d** The occluded EIA (arrow head) was atrophied. Arrow shows EIV. CFA: common femoral artery, EIA: external iliac artery, EIV: external iliac vein, SFA: superficial femoral artery, VR: volume rendering

**Fig. 2:**
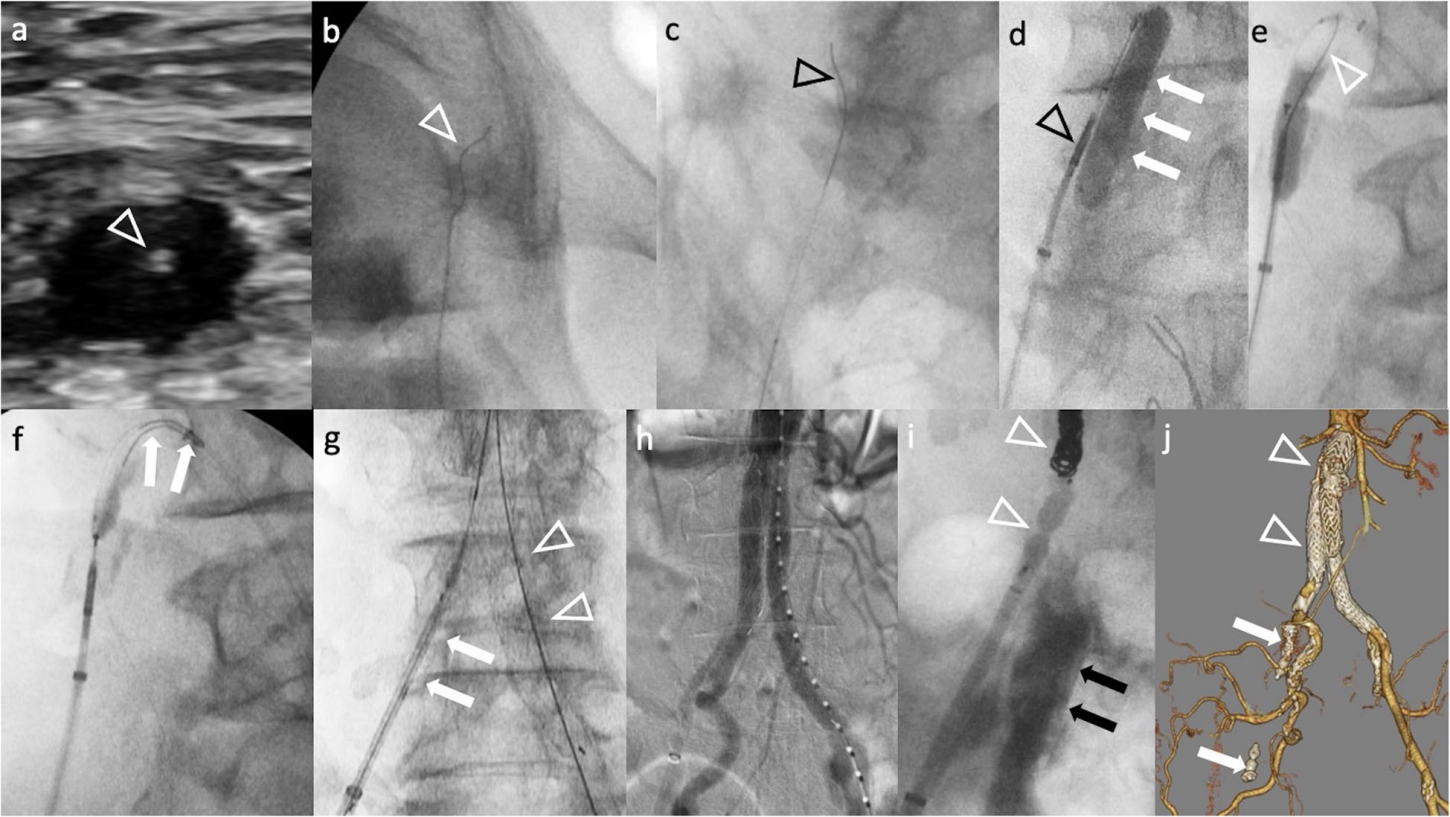
Procedure detail. **a** The occluded right CFA was punctured under ultrasound guidance (arrowhead: tip of the needle), **b** A guidewire (arrow heads) was advanced via the occluded right CFA, **c** A guidewire was advanced to the subintimal space of the right CIA, **d** The OUTBACK reentry device in the subintimal space (arrow head) was directed to an angioplasty balloon located at the lumen of the right CIA (arrows), **e** Insertion of the wire (arrowhead) into the punctured balloon, **f** Retracting the punctured balloon with the wire established a through and through wire, **g** Main body graft (arrowheads) which was delivered via left iliac artery was deployed and then the contralateral limb (arrows) was delivered via right iliac artery, **h** Completion angiogram shows a successful exclusion of the AAA, **i** Embolization of the tract was performed using coils and a plug (arrowheads) where a 12F sheath was advanced. A leakage of contrast media (arrows) indicates vessel disruption, **j** Post-operative CT shows implanted endograft (arrowheads), coils and plugs (arrows), and preserved collateral channels. AAA: abdominal aortic aneurysm, CFA: common femoral artery, CIA: common iliac artery

The case was an 82-year-old man who had a 50 mm infrarenal AAA (Fig. 1). The patient presented with a history of above the knee amputation of the right leg due to gangrene from Buerger’s Disease. CTA demonstrated extensive occlusion of the right EIA, CFA and SFA. As the collateral circulation originating from the right hypogastric artery fed the amputated right leg, no ischemic symptoms were observed in the leg. Since the patient was octogenarian, he requested an endovascular treatment. If a revascularization was to be performed, a concurrent FP bypass surgery would be required due to the occluded proximal SFA and profunda femoris artery (PFA). However, because of the absence of ischemic symptoms in his right leg, it was decided that EVAR be performed using the occluded vessel as a pathway to deliver the endograft, without revascularizing the vessel (Fig. 2). The occluded right CFA was punctured under ultrasound guidance using 21G needle (Medikit Co, Tokyo, Japan). A 0.014 in. guidewire (Command, Abbott, IL, USA) was inserted into the needle, and was then advanced to the level of the inguinal ligament. To improve the backup force, the needle was replaced with a microcatheter (Prominent Neo 2, Tokai Medical Products, Aichi, Japan). Under the support of the microcatheter, the guidewire was successfully advanced to the proximal ostium of the right EIA. Due to the impossibility of penetrating the hard proximal cap of the occluded EIA, a bidirectional approach was employed instead. A 6F guiding sheath (45 cm Destination, Terumo Corporation, Tokyo, Japan) was advanced from the left CFA to the right CIA. An antegrade penetration of the right EIA cap was also difficult, with the wire (0.014 in. Astato XS 9–40, Asahi Intec, Nagoya, Japan) going outside of the vessel easily. Intravascular ultrasound (Eagle Eye Platinum ST, Philips Healthcare, Eindhoven, Netherlands) located at the left CIA confirmed that the guidewire advanced from the occluded right CFA had entered the subintimal space of the right CIA. For re-entry of the guidewire into the true lumen, an OUTBACK ELITE Re-Entry catheter (Cordis, CA, USA) was employed. A semi-compliant angioplasty balloon (Sterling, 8–40 mm, Boston Scientific, MA, USA) was located at the right CIA, which was targeted by the re-entry catheter. A puncture of the inflated balloon was successfully performed using the re-entry device, followed by insertion of a guidewire into the punctured balloon. Retracting the punctured balloon into the sheath advanced from the left CFA while inserting the guidewire from the right CFA successfully established a through and through wire between the left and right CFAs. A 12Fr sheath (DrySeal Flex Introducer Sheath, WL Gore, Flagstaff, AZ, USA) was inserted from the right CFA. A 16 Fr Dryseal sheath for a main body graft was inserted from the left CFA. A conventional EVAR procedure was subsequently performed using an EXCLUDER AAA Endoprosthesis (WL Gore, Flagstaff, AZ, USA). A final angiogram showed the excluded aneurysm without any endoleak. Since there was an antegrade arterial flow into the right EIA which was used for the delivery of the 12F sheath, embolization was performed in order to occlude the channel. During the retraction of the 12F sheath, a leakage of contrast media was identified at a disrupted portion of the EIA. Detachable coils and plugs were used to embolize the channel [Interlock coils: 6 mm, 4 mm (Boston Scientific, MA, USA); AVP-2: 8 mm, 10 mm (Abbott, IL, USA)]. A post-operative CT confirmed that the aneurysm had been successfully excluded, while preserving the collateral vessels from the right hypogastric artery.

## Discussion

By using a direct puncture technique of the occluded CFA, we enabled EVAR for an AAA with unilateral iliac access occlusion extending to the SFA/PFA. The aorto-uni-iliac system, a possible option in a case with hostile iliac access, could compromise the arterial flow of the right hypogastric artery, which is an important collateral source of the right limb, leading to critical ischemia of the limb.

To introduce the endograft via an occluded access involving the CFA, the authors previously reported a technique where an angioplasty balloon was advanced distally to the occluded CFA via the occluded iliac artery from the contralateral CFA (Ichihashi et al. 2020). The balloon was then punctured under fluoroscopy consequently establishing a through and through wire between the bilateral CFAs. The technique, however, cannot be applied if the balloon cannot be delivered in an antegrade fashion via the occluded iliac artery down to the groin. In the present case, a proximal cap of the EIA occlusion was extremely hard. Using a guidewire with a high tip load can risk perforating the vessel. Direct puncture of the occluded CFA could be an effective alternative measure to deal with this situation. Kawasaki et al. reported on the efficacy of puncturing occluded vessels for peripheral vascular interventions (Kawasaki et al. 2021). While ultrasound guidance was used in the present case for the vessel puncture, the puncture can be performed under fluoroscopy in a case of heavily calcified wall (Kawasaki et al. 2021).

## Conclusions

AAA with access vessel occlusions involving the CFA was successfully managed with the direct puncture technique of the occluded CFA. The technique is simple, safe, and can broaden the indication of EVAR for AAA to include cases with hostile access.

## Data Availability

Not applicable.

## Abbreviations

AAA: Abdominal aortic aneurysm
CFA: Common femoral artery
CIA: Common iliac artery
EIA: External iliac artery
EVAR: Endovascular aneurysm repair
PFA: Profunda femoris artery
SFA: Superficial femoral artery
